# Time-course metabolic profiling in alfalfa leaves under *Phoma medicaginis* infection

**DOI:** 10.1371/journal.pone.0206641

**Published:** 2018-10-29

**Authors:** Qin Fan, Rebecca Creamer, Yanzhong Li

**Affiliations:** 1 State Key Laboratory of Grassland Agro-ecosystems, Key Laboratory of Grassland Livestock Industry Innovation, Ministry of Ministry of Agriculture and Rural Affairs, Engineering Research Center of Grassland Industry, Ministry of Education, College of Pastoral Agriculture Science and Technology, Lanzhou University, Lanzhou, Gansu Province, China; 2 Gansu University of Chinese Medicine, Lanzhou, Gansu Province, China; 3 New Mexico State University, Las Cruces, New Mexico, United States of America; 4 Institute of Grassland Research, Chinese Academy of Agricultural Sciences (CAAS), Hohhot, China; Fujian Agriculture and Forestry University, CHINA

## Abstract

Information on disease process and pathogenicity mechanisms is important for understanding plant disease. Spring black stem and leaf spot caused by the necrotrophic pathogen *Phoma medicaginis* var. *medicaginis* Malbr. & Roum causes large losses to alfalfa. However, till now, little is known about alfalfa-*P*. *medicagnis* interactions and the pathogenicity mechanisms of the pathogen. Here, alfalfa inoculated with *P*. *medicaginis* was subjected to GC-MS based metabolic profiling. The metabolic response in *P*. *medicaginis*-inoculated and mock-inoculated alfalfa leaves was assessed at 2, 4, 6, 8, 12, 16, 20, 24, 26 and 28 days post inoculation. In total, 101 peaks were detected in the control and inoculated groups, from which 70 metabolites were tentatively identified. Using multivariate analysis, 16 significantly regulated compounds, including amino acids, nitrogen-containing compounds and organic acids, polyols, fatty acids, and sugars were tentatively identified (Variable importance values, VIP>1.0 and *p <*0.05). Among these metabolites, the levels of malate, 5-oxoproline, palmitic acid and stearic acid were increased significantly in *P*. *medicaginis*-infected alfalfa leaves compared to the controls. In contrast, the levels ofγ-aminobutyric acid and 2-pyrrolidinone were significantly decreased in infected leaves compared to the controls. Further metabolic pathway analysis of the 16 significantly regulated compounds demonstrated that glycolysis, the tricarboxylic acid cycle, and β-oxidation of fatty acids were significantly induced in the alfalfa leaves at later stages of *P*. *medicaginis* infection. The strong induction of tricarboxylic acid cycle pathways at later infection stages caused by the pathogen may induce senescence in the alfalfa leaves, leading to plant death. However, intermediate metabolites of these metabolic pathways, and inositol phosphate, glutathione, the metabolic pathways of some amino acids accumulated rapidly and strongly at early stages of infection, which may enhance the ability of alfalfa to resist necrotrophic *P*. *medicaginis* disease. Understanding metabolic pathways is essential for understanding pathogenesis.

## Introduction

Alfalfa (*Medicago sativa* L.) is grown worldwide as a perennial high quality forage [1]. Alfalfa acreage in China has rapidly increased since the Chinese government initiated a program to promote the milk and alfalfa industries in 2012 [2]. The alfalfa acreage in 2015 was 4.7 million ha, which is planned to increase to more than 133 thousand ha in 2020 [3]. However, diseases limit alfalfa production [4]. Spring black stem and leaf spot caused by *Phoma medicaginis* var. *medicaginis* Malbr. & Roum commonly occurs in China [5–7], North America, Europe, and Africa [8–10], causing yield losses, decreasing forage quality [11], and affecting the health of livestock [12]. Previous studies showed that one variety, Aohan, bred in Inner Mongolia, China was highly resistant among the 70 alfalfa varieties tested from many counties including China, America, France, Australia, and Canada. [13,14]. In two moderately resistant alfalfa genotypes, the leaves delayed spore germination, penetration, development of mycelium, pycnidia formation, and symptom development compared with leaves from susceptible alfalfa genotypes [11]. In recent studies of mechanisms of pathogenesis and disease-resistance, we showed that *P*. *medicaginis* decreased the transfer rate and capture efficiency of photosynthetic electrons, the amount of non-photochemical quenching (qN), and the carboxylation efficiency (CE) in alfalfa leaves [15]. Activity of the antioxidant enzymes, superoxide dismutase (SOD), catalase (CAT), and phenylalanine ammonia 1yase (PAL), increased earlier and to higher levels in resistant alfalfa varieties than in susceptible alfalfa varieties [13]. During infection of alfalfa with *P*. *medicaginis*, the fungitoxic phytoalexins, isoflavonoids such as medicarpin, sativan, formononetin 7-*O*-glucoside, and malonylated formononetin 7-*O*-glucoside increased in alfalfa leaves infected by *P*. *medicaginis* [16–18], and the activity of enzymes, and corresponding transcript level, involved in isoflavonoid biosynthesis including isoflavone reductase (IFR), PAL, and chalcone synthase (CHS) were also upregulated [16]. β-1, 3-glucanase was stimulated in the transcription of leaf tissues and cell suspensions of alfalfa [19]. Currently, little is known about the disease process and mechanism of pathogenesis for *P*. *medicaginis* on alfalfa, information which is important for the development of novel disease-control strategies and breeding resistant varieties. In this study, we analyzed the expressions of metabolites in *P*. *medicaginis*-inoculated and mock-inoculated alfalfa plant leaves using GC-MS. The up- or down-regulated metabolites were further analysed for their metabolism pathways and a complex metabolic network was developed. This complex metabolic network should extend our understanding of the mechanisms controlling *P*. *medicaginis* pathogenicity and alfalfa tolerance to this pathogen.

## Materials and methods

### Experimental design

The strain of *P*. *medicaginis* var. *medicaginis*, LYZ00164 (Genbank accession KP207577) was isolated from alfalfa leaves collected from the field in Gansu province, China. The isolate was cultured on potato dextrose agar (PDA) media at 25 °C without light in an incubator for 3 weeks. A spore suspension was prepared with sterile distilled water, and the number of spores in the suspension was adjusted to 1–1.5×10^6^ spores⋅mL^−1^ with a hemocytometer [13]. Seeds of the susceptible alfalfa cultivar, Derby [15] were sown in 70 pots (6 seeds per pot) containing sterilized soil, and grown inside a greenhouse set at 25/15 °C (day/night) for two months. Thirty pots of seedlings were spray inoculated with a spore suspension of *P*. *medicaginis* using an atomizer, and the seedlings were covered with black plastic bags for 48 h to promote infection, then, grown without plastic bags in the greenhouse. Thirty additional pots of seedlings were sprayed with sterile water as healthy controls, and 10 pots of seedlings were retained for positive controls to observe symptoms and disease development. Symptoms were observed after inoculation. All plants were grown under the same conditions. Fully expanded leaves at the same development stage were harvested from the *P*. *medicaginis*- or water-inoculated plants at 2, 4, 6, 8, 12, 16, 20, 24, 26 and 28 days post inoculation (dpi). Three pots with six plants each were sampled at each sampling date. The harvested leaves were quickly frozen with liquid nitrogen and stored at -80°C until use.

### Metabolite extraction, determination and identification

Leaf samples (30 mg each, n = 3) were pulverised individually in mortars, and extracted with MeOH: H_2_O (3 × 0.5 mL, 4:1, v/v) and 75 μL ribitol (2 μg/μL^–1^, Shanghai Yuanye Biotechnology Co. LTD), which was used as an internal standard. MeOH soluble fractions were dried individually with nitrogen and each sample was gently mixed with 45 μL methoxyamine hydrochloride (20μg/μL^–1^, J&K Scientific Ltd.). The mixtures were incubated at 30 °C for 90 min followed by addition of 45 μL N,O-bis-(trimethylsilyl)-trifluoroacetamide (J&K Scientific Ltd.) to each sample and incubation at 70 °C for 30 min. Each sample was diluted with 90 μL hexane prior to injection as described previously [20].

An Agilent 7890A/5975C gas chromatograph-tandem mass spectrometer equipped with an autosampler 7693 (Agilent Technologies, America) was used for GC-MS detection. The detection conditions were as follows: ion source temperature at 230 °C, electron beam at 70 eV, Agilent 19191S-433 column with 30 m × 0.25 mm internal diameter and 0.25 μm film thickness, helium gas flow rate at 1 ml/min^-1^, splitless injection, and mass range at m/z 50–650. Constituents were determined under the following conditions: 80 to 240 °C at 6 °C per min, 240 to 280 °C at 10 °C per min, and 280°C for 10 min. The injection temperature was 270 °C. Three replication for GC-MS.

Compounds in leaves were tentatively identified with Automated Mass Spectral Deconvolution and Identification System (AMDIS), our in-house mass spectra library and the standard mass spectral database (National Institute of Standards and Technology, NIST, version 11.0). The signal/noise (S/N) of the tentatively identified compound was higher than 10 in the AMDIS and the compound shared greater than or equal to 80% similarity with the known compounds in the database, indicating that the metabolite identification was reliable [21,22].

### The identification of significantly different metabolites and their metabolic pathway construction

The amounts of tentatively identified compounds were normalised with the mean value of the internal standard. The normalised data were imported into SIMCA14.1 (Umetrics, Sweden) for further multi-variate statistical analysis, including principal component analysis (PCA) and orthogonal partial least-squares discriminant analysis (OPLS-DA). The Q^2^ value is a prediction error obtained through OPLS-DA analyses, and values greater than or equal to 0.5 are generally considered to have good predictive capability [23]. To validate OPLS-DA models, 200 random permutation tests were performed. The variable importance values (VIP) were obtained through OPLS-DA analyses. When the VIP value of a metabolite exceeded 1.0, this metabolite was considered to be a potentially regulated metabolite and its variables were further analysed using a two-way analysis of variance. When the *p* value was less than 0.05, this metabolite was considered to be a significantly regulated metabolite compared to the controls [24]. The significantly regulated metabolites were analysed using the MetaboAnalyst platform (www.metaboanalyst.ca/). The final metabolic network was established according to KEGG (http://www.genome.jp/kegg/) [25]. A heat map was drawn with Mev 4.8. Student’s t test was used for comparison between groups at the same time point.

## Results

### Symptoms and disease development

**Fig 1:**
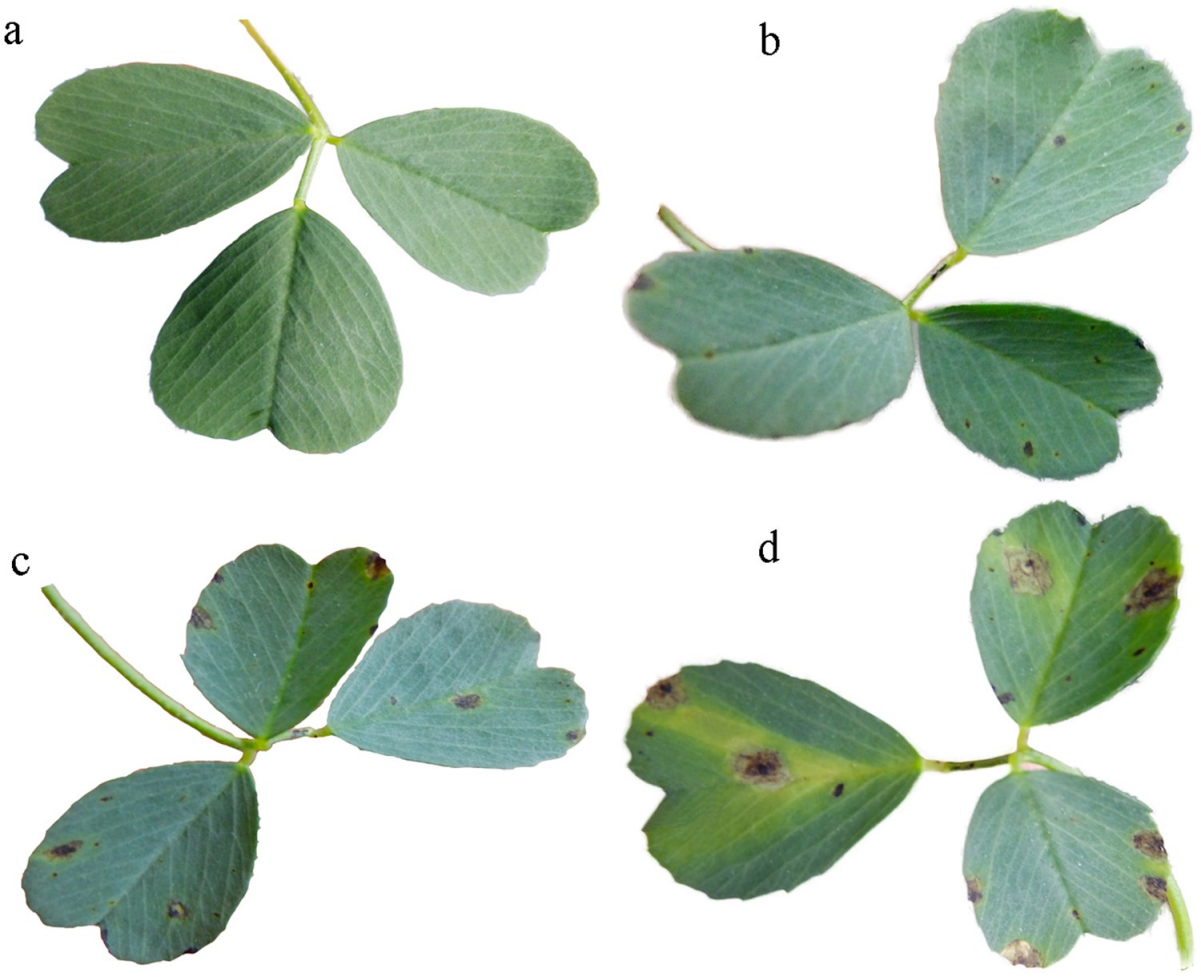
Symptoms on alfalfa inoculated with *Phoma medicaginis* var. medicaginis. The symptoms observed at 8 d (b), 19 d (c) and 26 d (d) after inoculation, and without inoculation (controls) (a).

Symptoms were observed as small black spots on inoculated leaf blades on the sixth day post inoculation (Fig 1b). Subsequently, lesions enlarged and coalesced (Fig 1c and 1d), while no such symptoms appeared on the leaf blades of controls (Fig 1a).

### Metabolite profiles and metabolite response to pathogen stress

In total, 101 peaks were detected by GC-MS. From the peaks, 70 unique compounds were tentatively identified, including 23 carbohydrates and alcohols, 23 amino acids, 5 nitrogen-containing compounds, 10 organic acids, 5 lipids, 4 other compounds (Table 1).

**Table 1:** Identification of 70 metabolites from the control and inoculated leaves and the determination of 16 significantly regulated metabolites associated with *P*. *medicaginis* infection.

No.	Compound	RT (min)	Characteristic fragment	Similarity	Inoculated group	Control group	*p*	VIP
1	Glycine^a^^b^	10.452	102	90	0.025±0.001	0.035±0.002	0.001	1.199
2	Leucine^a^^b^	14.581	158	95	0.270±0.016	0.362±0.020	0.001	1.214
3	Isoleucine^a^^b^	15.124	158	92	0.475±0.030	0.768±0.050	0.001	1.426
4	Tyrosine^a^^b^	27.909	218	96	0.119±0.011	0.228±0.013	0.001	1.577
5	Methionine^a^^b^	20.166	176	94	0.018±0.005	0.066±0.006	0.001	1.532
6	5-oxoproline^a^^b^	20.233	156	95	0.161±0.011	0.121±0.006	0.001	1.146
7	Lysine^a^^b^	23.843	156	96	0.070±0.004	0.093±0.006	0.001	1.088
8	GABA^a^^b^	15.201	102	90	0.149±0.016	0.398±0.055	0.001	1.204
9	2-pyrrolidinone^a^	11.037	142	92	0.029±0.003	0.050±0.002	0.001	1.592
10	Myoinositol^a^	28.399	217	85	0.155±0.016	0.263±0.018	0.001	1.258
		29.109	217	80				
11	Inositol 1,3,4,5,6-pentakisphosphate^a^	30.537	554	85	0.197±0.016	0.380±0.047	0.001	1.142
12	Sucrose^a^	37.348	361	94	1.919±0.197	3.285±0.252	0.001	1.285
13	Malate^a^	19.653	233	91	1.585±0.058	0.940±0.082	0.001	1.556
14	Malonic acid^a^	12.828	233	95	0.117±0.014	0.381±0.041	0.001	1.549
15	Palmitic acid^a^^b^	29.374	313	95	0.530±0.023	0.339±0.022	0.001	1.442
16	Stearic acid^a^^b^	32.325	341	99	0.537±0.028	0.327±0.030	0.001	1.321
17	Acetic acid, hydroxy-^a^	9.232	205	92	0.014±0.000	0.012±0.000	0.001	0.901
18	Pyruvic acid ^a^	9.601	217	93	0.014±0.001	0.011±0.001	0.001	0.95
19	Alanine^a^^b^	10.025	116	93	0.276±0.013	0.297±0.017	0.163	0.762
20	Ethanedioic acid^a^	10.867	190	94	0.016±0.001	0.018±0.001	0.004	0.834
21	α-Aminobutyric acid^a^	11.949	130	88	0.022±0.002	0.022±0.002	0.907	0.776
22	Urea^a^	12.250	261	86	0.034±0.002	0.028±0.002	0.038	0.989
23	beta-Alanine^a^	12.382	102	86	0.127±0.013	0.224±0.030	0.001	0.914
24	Valine^a^^b^	13.174	144	89	1.094±0.088	1.057±0.083	0.408	0.820
25	Benzoic acid^a^	13.792	179	80	0.081±0.009	0.120±0.010	0.001	0.986
26	Phosphate^a^	14.658	299	89	0.144±0.018	0.111±0.010	0.024	0.817
27	Glycerol^a^	14.724	205	93	0.327±0.022	0.313±0.014	0.522	0.768
28	Succinate^a^	15.495	247	85	0.995±0.124	0.888±0.060	0.108	0.772
29	Glyceric acid^a^	16.068	189	92	0.057±0.003	0.061±0.003	0.069	0.756
30	Fumarate^a^	16.270	245	83	0.020±0.001	0.022±0.001	0.086	0.735
31	Pipecolic acid^a^	16.691	156	91	0.053±0.009	0.039±0.002	0.076	0.884
32	Serine^a^^b^	16.805	204	91	1.106±0.066	1.122±0.065	0.747	0.723
33	Threonine^a^^b^	17.420	218	95	0.670±0.049	0.670±0.047	1	0.799
34	2-Piperidone, amino-^a^	18.833	128	80	0.045±0.004	0.051±0.004	0.004	0.827
35	2,2-hydroxymalonate ^a^	19.028	308	95	0.058±0.003	0.072±0.003	0.001	0.801
36	Aspartic acid^a^^b^	20.256	232	94	0.737±0.130	0.487±0.022	0.022	0.906
37	Threonic acid^a^	21.248	292	95	0.147±0.008	0.169±0.006	0.001	0.946
38	Threo-2-pentulose^a^	21.703	306	80	1.575±0.098	1.369±0.073	0.03	0.864
39	Ornithine^a^^b^	22.122	142	91	0.260±0.032	0.297±0.027	0.112	0.801
40	Glutamic acid^a^^b^	22.235	246	94	0.368±0.017	0.360±0.020	0.742	0.517
41	Phenylalanine^a^^b^	22.335	192	94	0.792±0.070	0.917±0.074	0.018	0.886
42	Arabitol^a^	22.938	147	80	0.108±0.005	0.119±0.004	0.05	0.812
43	Asparagine^a^^b^	23.308	231	95	4.276±0.407	4.110±0.323	0.467	0.799
44	Arabinose^a^	23.586	217	90	0.142±0.007	0.126±0.003	0.004	0.983
45	α-Aminoadipic acid^a^	24.027	260	89	0.052±0.004	0.042±0.003	0.001	0.885
46	Phosphoric acid, 2,3- hydroxypropyl ester^a^	25.049	357	84	0.063±0.005	0.071±0.004	0.147	0.827
47	2-keto-1-guconic acid^a^	25.193	292	92	0.111±0.005	0.114±0.004	0.526	0.679
48	Fructofuranose^a^	25.906	217	85	0.294±0.035	0.390±0.054	0.033	0.796
49	Citrate^a^	26.062	273	94	0.436±0.030	0.515±0.031	0.05	0.811
50	Pinitol^a^	26.412	217	90	8.838±0.361	8.151±0.154	0.017	0.839
51	Adenine^a^	26.701	264	92	0.077±0.005	0.078±0.004	0.702	0.619
52	Fructose^a^	27.098	217	93	0.524±0.057	0.710±0.070	0.004	0.871
53	Sorbose^a^	27.279	217	89	0.628±0.070	0.899±0.100	0.001	0.895
54	Galactopyranoside^a^	27.506	204	88	1.529±0.106	2.139±0.054	0.002	0.896
55	Galactose^a^	27.542	319	86	0.940±0.120	0.946±0.067	0.001	0.652
		27.765	319	90				
56	Histidine^a^^b^	27.627	254	84	0.206±0.027	0.262±0.023	0.005	0.874
57	Ethyl-glucopyranoside^a^	28.138	204	84	0.404±0.071	0.497±0.056	0.003	0.725
58	Glucose^a^	28.844	204	93	0.180±0.017	0.291±0.045	0.005	0.881
		31.282	319	87				
		34.968	204	91				
59	Gluconic acid^a^	29.110	333	80	0.106±0.005	0.132±0.004	0.001	0.925
60	Tryptophan^a^^b^	32.253	202	93	0.324±0.029	0.343±0.023	0.223	0.763
61	Lactulose^a^	33.610	204	82	0.047±0.002	0.049±0.002	0.472	0.812
62	Glyceryl-glycoside^a^	33.813	204	90	0.175±0.012	0.172±0.004	0.748	0.618
63	Glucuronic acid^a^	34.502	204	80	0.131±0.004	0.124±0.004	0.118	0.834
64	Uridine^a^	35.220	217	90	0.189±0.008	0.164±0.007	0.001	0.829
65	2-Monopalimitoylglycerol^a^	36.077	129	88	0.043±0.001	0.041±0.002	0.254	0.719
66	Monopalmitin^a^	36.399	371	81	0.690±0.062	0.951±0.053	0.001	0.968
67	Turanose^a^	36.585	361	82	0.074±0.003	0.082±0.003	0.035	0.917
68	2-Monostearin^a^	37.922	129	80	0.077±0.004	0.077±0.003	0.817	0.578
69	Monostearin^a^	38.317	399	86	0.163±0.006	0.188±0.007	0.003	0.915
70	Gentiobiose^a^	39.690	204	83	0.035±0.002	0.039±0.002	0.063	0.775

RT = retention time of compound.

^a^ Identified by NIST data base.

^b^ Identified by our in-house mass spectra library.

Data from inoculated and control groups was the average of the sum of the normalised data of the compound at each time point, which presented as Mean±SE (n = 3). The data were evaluated by a two-way analysis of variance, through which the *p* value was calculated. The data were determined at 2, 4, 6, 8, 12, 16, 20, 24, 26 and 28 dpi, dpi (day post inoculation). The variable importance values (VIP) were obtained through OPLS-DA analyses.

**Fig 2:**
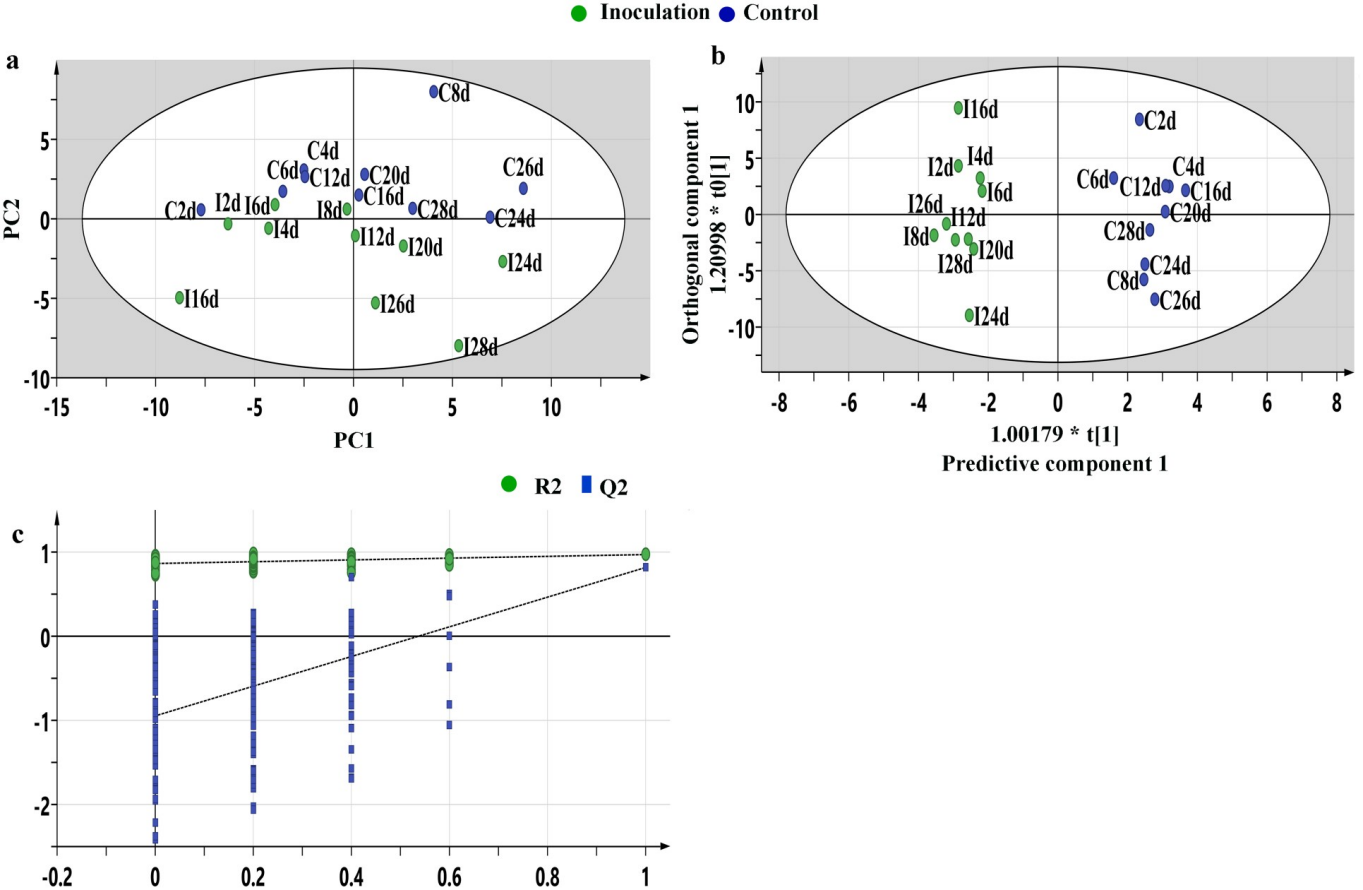
Multivariate statistical analysis of control samples and infected samples. Principal component analysis (PCA) score plot for the inoculated samples (green) and control samples (blue) collected at 2, 4, 6, 8, 12, 16, 20, 24, 26 and 28 days, PC1 is 35.3% and PC2 is 17.2% (a); Orthogonal partial least-squares discriminant analysis (OPLS-DA) score plot (1+4 components), R^2^X_(cum)_ = 0.739, R^2^Y_(cum)_ = 1.000, Q^2^ = 0.817), R^2^X_(cum)_ and R^2^Y_(cum)_ are the cumulative modelled variations in the X and Y matrices, respectively, and Q^2^ value is a prediction error (b); Validation plots of the orthogonal partial least-squares discriminant analysis (POLS-DA) models acquired through 200 permutation tests for the control vs. inoculated groups. T [1] = scores for predictive component 1, to [1] = scores for orthogonal component 1. The ellipse shows the 95% confidence interval using Hotelling T^2^ statistics. R and Q were obtained after OPLS-DA permutation tests (n = 200), R^2^ intercept was 0.861, Q^2^ intercept was -0.933 (c).

The R^2^X (cum) value of the PCA model is 0.529, as shown in Fig 2a. The model could not be used to distinguish between control and pathogen-inoculated groups, however, the OPLS-DA results clearly placed the control and pathogen treatments in two distinct groups. The samples collected from control and pathogen-inoculated groups all fell inside the 95% Hotelling T^2^ ellipse (Fig 2b). The R^2^Y_(cum)_ and Q^2^ values of this model were 1.000 and 0.817, respectively. Validation plots showed R2 and Q2 values from the permuted analysis (bottom left) are lower than the corresponding original R2 and Q2 values (top right), indicating a good fit for the model (Fig 2c).

**Fig 3:**
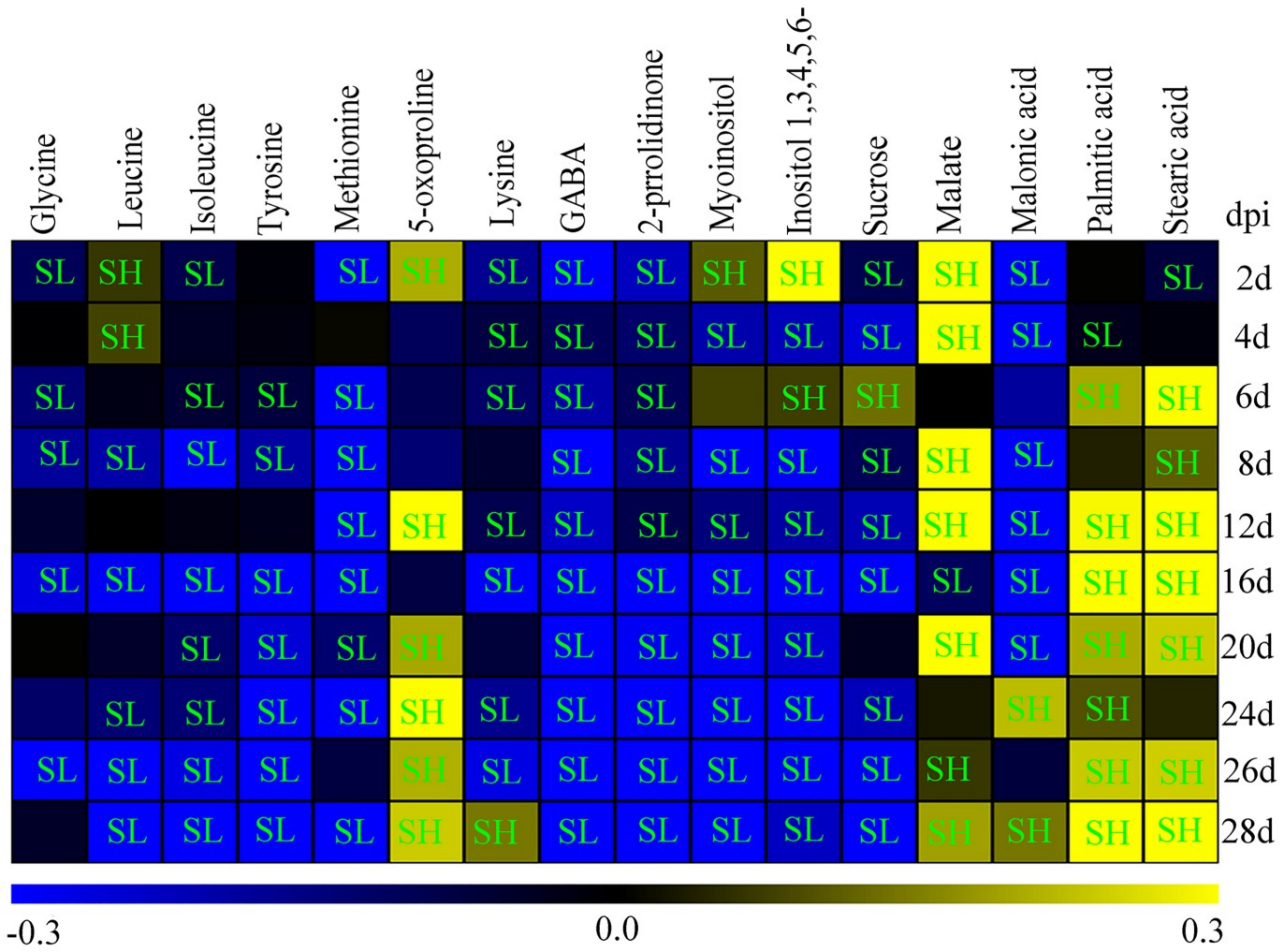
Heat map of significantly regulated metabolites in the alfalfa leaves between control and inoculated groups. Metabolite levels in the leaves were shown as log_10_ ratios of values from infected versus uninfected groups at 2, 4, 6, 8, 12, 16, 20, 24, 26 and 28 days post inoculation (dpi). Yellow color shows upregulated compound levels, and the blue color indicates downregulated levels. Level of compounds in the inoculated leaves was significantly lower (SL) or significantly higher (SH) than in the control leaves. Inositol 1,3,4,5,6- is inositol 1,3,4,5,6-pentakisphosphate. Data were evaluated by student’s t test and *p*<0.05 has significant difference.

A total of 16 significantly regulated metabolites (VIP > 1 and *p* < 0.05) associated with the pathogen infection were identified using the OPLS-DA model. These metabolites included eight amino acids (glycine, leucine, isoleucine, lysine, tyrosine GABA, 5-oxoproline, and methionine), one nitrogen-containing compound (2-pyrrolidinone), two organic acids (malonic acid and malate), two polyols (myoinositol and inositol 1,3,4,5,6-pentakisphosphate), one sugar (sucrose) and two fatty acids (palmitic acid and stearic acid) (Fig 3).

Several amino acids, such as glycine, leucine, isoleucine, methionine and tyrosine, and sucrose, myoinositol and inositol 1, 3, 4, 5, 6-pentakisphosphate were downregulated in the *P*. *medicaginis*-inoculated leaves compared to the mock-inoculated leaves after 6 dpi. In contrast, the level of lysine in the *P*. *medicaginis*-inoculated leaves was gradually upregulated 39.1% by 28 dpi.

Some compounds showed a dramatic increase in the *P*. *medicaginis*-inoculated leaves compared with mock-inoculated leaves; for example, the level of 5-oxoproline in the *P*. *medicaginis*-inoculated leaves reached a maximum level of 137.9% by 24 dpi, falling to 74.2% by 28 dpi. Malate dramatically increased by 352.4% at 4 dpi. The levels of palmitic acid and stearic acid reached a maximum level of 266.8% and 291.4%, at 28 dpi, respectively.

Some compounds showed a large decrease in the *P*. *medicaginis-*inoculated leaves compared with the mock-inoculated leaves. The levels of GABA (γ-Aminobutyric acid) and 2-pyrrolidinone decreased over time compared with the control groups, and the levels of malonic acid significantly decreased at early infection stages (*p*<0.05), but increased by 67.2% at 24 dpi and 38.1% at 28 dpi (t test, *p*<0.05).

Metabolites related to glycolysis and the TCA cycle were significantly induced at later infection stages (t test, *p*<0.05). For example, at 28 dpi, pyruvic acid increased 1.69 fold, asparagine increased 1.41 fold, succinate increased 1.90 fold, fumarate increased 1.37 fold, and citrate increased by 2.16 fold compared with the control groups (Table 2).

**Table 2:** Fold change values from pathogen-inoculated versus control groups at each time points.

Metabolite	Fold change									
	2dpi	4dpi	6dpi	8dpi	12dpi	16dpi	20dpi	24dpi	26dpi	28dpi
Pyruvic acid	1.14	1.45*	1.36*	1.20**	1.30	0.59*	1.48*	1.46*	0.97	1.69**
Asparagine	0.81**	1.26**	0.89	0.93	1.25*	0.42**	1.46**	1.13	0.84	1.41**
Succinate	0.77	0.54**	0.80	0.86	1.03	0.39**	1.43**	1.29**	1.41**	1.90**
Fumarate	0.92	0.62	1.08	0.45	0.94	0.41	1.00	1.33	1.38*	1.37**
Citrate	0.77**	0.44*	0.78	1.40**	0.98	0.22**	1.27**	0.54**	1.20**	2.16**

Fold change is the ratio of data from inoculated versus control groups at 2, 4, 6, 8, 12, 16, 20, 24, 26 and 28 days post inoculation (dpi). Data were evaluated by student’s t test and indicated by asterisks (*p<0.05 or **p<0.01).

### Metabolic pathways

**Fig 4:**
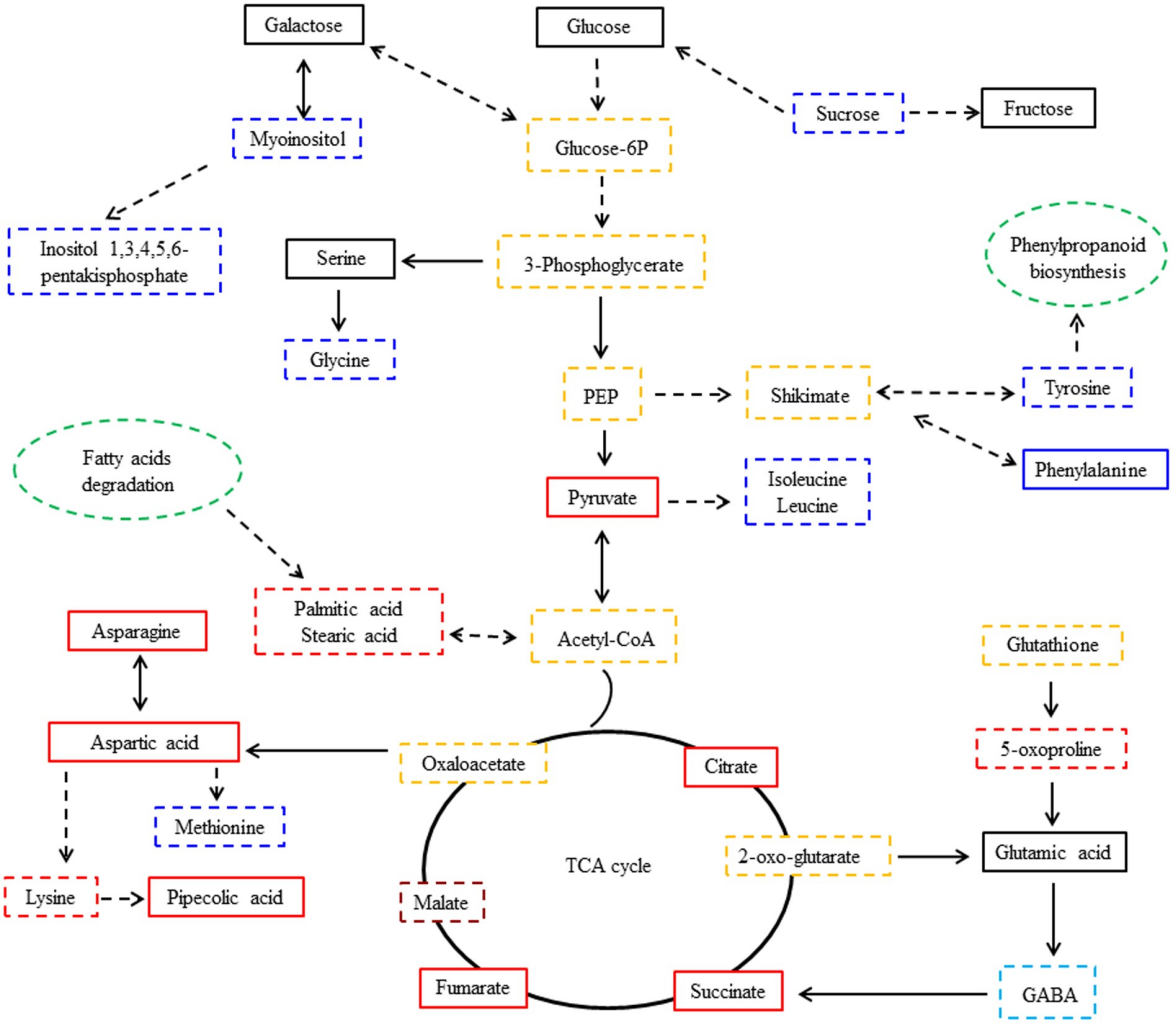
Metabolic pathway network of responsive metabolites in alfalfa leaves infected with *P*. *medicaginis*. The black, red, blue, light blue and crimson full and dotted box indicates that metabolites were detected by GC-MS from the alfalfa leaves, and the dotted box presents significant regulated metabolites in the inoculated leaves compared with the controls (VIP>1.0 and *p*<0.05). Yellow dotted box indicates that the metabolites were not detected by GC-MS from the alfalfa leaves, but were involved in the corresponding pathway. Green dotted ovals indicates that the metabolic pathway was associated with the regulated metabolites. (VIP, the variable importance for the projection; PEP, phosphoenolpyruvate; TCA cycle, tricarboxylic acid cycle). Red color indicates that the compound was significantly upregulated at later infection stages. Blue color indicates that the compound was significantly downregulated at later infection stages. Light blue color indicates that the compound was significantly downregulated over time. Crimson color indicates that the compound was almost significantly upregulated over time.

Of the 16 compounds regulated with infection, 14 compounds were associated with 30 primary metabolic pathways (S1 Table). These pathways include metabolism of sugar and alcohol, metabolism of amino acids and organic acids, metabolism of fatty acids and glutathione (S1 Table), of which glycolysis, amino acids, inositol phosphate, TCA cycle, fatty acids, and glutathione are most important (Fig 4). The pathway of 2-pyrrolidinone and malonic acid metabolites were not found to belong to a specific defined metabolic pathway.

## Discussion

During antagonistic interactions between plants and pathogenic fungi, the pathogens try to modulate plant carbon and nitrogen metabolism to ensure their proliferation and survival, while plants need nutrients and energy to synthesize phytoalexins, defense related protein, antioxidants and many other metabolites to resist pathogen infection [26].

As disease progressed, lesions enlarged and coalesced, leaves became yellow, and the levels of 16 metabolites in the infected plants were significantly changed compared to the non-infected plants during *P*. *medicaginis* colonization. These metabolites included amino acids (glycine, leucine, isoleucine, tyrosine, lysine, 5-oxoproline, methionine, and GABA), 2-pyrrolidinone and organic acids (malonic acid and malate), sucrose, alcohols (myoinositol and inositol 1,3,4,5,6-pentakisphosphate), and fatty acids (palmitic acid and stearic acid).

The levels of glycine, isoleucine, leucine and tyrosine in the *P*. *medicaginis*-infected alfalfa leaves decreased compared to the uninfected plants. These amino acids are not only the basic units of protein synthesis, but also enhance defense responses to biotic stresses including strengthening of plant cell walls, regulation of jasmonic acid (JA), and biosynthesis of phytoalexins. [27–30]. This suggests that the decrease in these amino acids levels in the infected leaves may promote the susceptibility of alfalfa to the pathogen infection.

As shown in Fig 4, 5-oxoproline and glutathione (GSH) are involved in the glutathione metabolism pathway; 5-oxoproline is a degradation product of glutathione. Upon *P*. *medicaginis* infection, 5-oxoproline levels significantly increased in the inoculated alfalfa leaves in different infection stages, but especially in the later infection (*p*<0.05). This suggests that the antioxidant system based on the glutathione-mediated detoxification pathway may be induced mostly later in infection. High accumulation of 5-oxoproline may increase plant resistance, and lower levels of the metabolite at early infection stages may help the pathogen colonize the plant. Previous studies showed that antioxidant enzyme activity was induced earlier and to higher levels in the resistant varieties of alfalfa than in the susceptible varieties [13]. These results reinforce the expectation that antioxidant compounds and enzymes play a synergistic role in plant disease resistance.

With *P*. *medicaginis* infection, lysine and its degradation product pipecolic acid were induced later and strongly in infected alfalfa leaves, compared with control leaves, which could be related to alfalfa susceptibility. Pipecolic acid was previously shown to induce systemic acquired resistance (SAR) [31]. Pipecolic acid was produced faster and higher in *Arabidopsis* leaves inoculated with the incompatible, HR-inducing *Psm avrRpm1* strain than in *Arabidopsis* leaves infected with the compatible *Psm* strain, and pipecolic acid accumulated to higher levels during later stages of the compatible interaction [32]. Therefore, later strong induction of lysine degradation in the infected alfalfa leaves may be a way for the pathogen to escape host resistance.

For some pathogens, they need to assimilate methionine or its derivatives from host tissue to support their growth, which is related to their pathogenicity. For instance, methionine auxotrophic mutants of the pathogens *Ustilago maydis* and *Fusairum graminearum*, showed only reduced pathogenicity on plant leaves and were still able to penetrate and colonize host tissues [33,34]. Under *P*. *medicaginis* infection, methionine levels decreased, which may be related to the assimilation of *P*. *medicaginis*. The pathogen may need to acquire sufficient amounts of methionine from alfalfa leaves to support its growth. Inhibiting the use of methionine by the pathogen can increase alfalfa resistance.

As shown in S1 Table and Fig 4, myoinositol (inositol) and inositol-1, 2, 3, 4, 5, 6-hexakisphosphate are involved in the inositol phosphate metabolism pathway. With *P*. *medicaginis* infection, myoinositol and inositol derivatives (Inositol 1, 3, 4, 5, 6-pentakisphosphate) were induced early but decreased significantly at later infection stages in the infected leaves (*p*<0.01), which suggested that ability of alfalfa to resist disease decreased. Conversely, early strong induction of myoinositol and inositol derivatives (Inositol 1, 3, 4, 5, 6-pentakisphosphate) may enhance alfalfa resistance. The amounts of myoinositol and inositol derivatives in resistant wheat cultivars (Sumai3) were higher than that in susceptible wheat cultivars (Roblin) [35].

In this research, malonic acid decreased in the infected leaves significantly at early infection stages (*p*<0.05), and accumulated at later infection stages. Malonic acid metabolism likely provides a carbon source for the survival of microorganisms [36,37]. Therefore, the changes of malonic acid in the alfalfa detected may have provided carbon for *P*. *medicaginis* to invade, increasing host susceptibility.

As shown in Fig 4, metabolism of sucrose relates to the glycolysis pathway; its end product is pyruvic acid (pyruvate), which was induced significantly at later infection stages (*p*<0.05) (Table 2). The decrease in sucrose levels may also be involved in promoting the glycolysis pathway in the later infection stages, except when it serves as a carbon source for fungal mycelium growth [5,11].

In this study, the TCA cycle intermediates malate, fumarate, succinate, and citrate all accumulated significantly in infected leaves at later infection stages (*p*<0.05) (Fig 3 and Table 2). Moreover, as shown in Fig 4, GABA and asparagine are synthesized by the TCA cycle, asparagine were induced strongly at late infection stages when compared with controls (Table 2). Asparagine is one of the major metabolic products in senescing leaves [38], and it could increase the *P*. *medicaginis* spore germination rate [39]. GABA also likely provided an extra source of translocated nitrogen for the growth of the fungus [40], 2-Pyrrolidinone (pyrrolidinone) is one important metabolites of GABA [41]. These results suggest that impaired photosynthesis of alfalfa leaves caused by the infection of *P*. *mecaginis* may lead to a very strong induction in respiration, TCA cycle and glycolysis in the infected leaves, which could compensate for reduced ATP to the host cells as a result of the impaired photosystem [15]. In addition, the decrease in GABA and 2-Pyrrolidinone in the infected leaves may relate to depletion of *P*. *medicaginis*, since these compounds may also provide nitrogen for the pathogen growth and survival. The induction of asparagine through the TCA cycle following *P*. *medicaginis* infection provides a rich nitrogen reservoir to support fungal growth in alfalfa and also facilitate pathogen-initiated host senescence. However, the strong induction of plant respiration by the pathogen may induce senescence leading to host plant death.

Palmitic acid involves fatty acid degradation pathways (S1 Table and Fig 4), the primary manner of fatty acid degradation is β-oxidation, which not only provides a large amount of energy and carbon sources needed for the life activities of plant, but also provides abundant fatty acids and carbon source for pathogen invasion and growth. Under *P*. *medicaginis* infection, genes in the octadecanoid pathway were induced significantly and accumulated more rapidly in a resistant *M*. *truncatula* accession than in a susceptible *M*. *truncatula* accession [30]. In our studies, large amounts of palmitic acid (C16) and stearic acid (C18) accumulated in later *P*. *medicaginis* infection stages, suggesting that the induction of β-oxidation in the later infection stages may have provided nutrition for *P*. *medicaginis* for invasion and growth, while the rapid accumulation of these two fatty acids at early infection stages may enhance plant resistance to the pathogen infection.

This research was carried out on a susceptible cultivar. We anticipate that a resistant cultivar would induce intermediate metabolites of glycolysis, TCA cycle and β-oxidation metabolism pathways, and inositol phosphate, glutathione, some amino acids metabolic pathways, such as glycine, leucine, isoleucine, tyrosine and lysine, rapidly and strongly at an early infection stage. When Kamphuis and coworkers compared susceptible and resistant cultivars of *M*. *truncatula* infected by *P*. *medicaginis*, they found expression of genes associated with disease resistance was induced to lower levels in a resistant cultivar later in infection than for a susceptible cultivar. Their experiment showed the greatest upregulation in *PR10* gene expression, lipoxygenase transcripts and isoflavone 2’-hydroxylase in the octadecanoid pathway from resistant plants earlier and to a higher level [30]. Our work was done with a different system at the metabolic level, however, we would anticipate that glycine, leucine, isoleucine, tyrosine, lysine, 5-oxoproline, myoinositol and inositol 1,3,4,5,6-pentakisphosphate, palmitic acid and stearic acid would likely be induced early and higher in resistant cultivars. These compounds and their related metabolic pathways could provide information for the identification and characterization of resistant cultivars.

## Conclusion

A detailed primary metabolic profile of the response of alfalfa leaves to *P*. *medicaginis* infection was presented. In the process of *P*. *medicaginis* colonization of the alfalfa leaves, glycolysis, TCA cycle and β-oxidation metabolism pathways in the infected leaves accumulated strongly at later infection stages, which may provide ATP, a N source and nutrition for *P*. *medicaginis* to colonize alfalfa leaves. Moreover very strong induction of the TCA cycle pathways by the pathogen at later infection stage may induce senescence in alfalfa leaves, leading to plant death. Intermediates metabolites of these metabolic pathways, and inositol phosphate, glutathione, some amino acids metabolic pathways, such as glycine, leucine, isoleucine, tyrosine and lysine, accumulated rapidly and strongly at an early infection stage may enhance the ability of alfalfa to resist necrotrophic *P*. *medicaginis*. The tentatively identified differential metabolites and related pathways may serve as potential markers for developing novel disease-control strategies and breeding resistant varieties.

## Supporting information

S1 Table Relevant pathways of 16 significant regulated metabolites. Relevant pathways based on MetaboAnalyst 4.0 analysis.
(DOCX)

S2 Table Amount of metabolites in control and inoculated groups at the different times. Amount of individual metabolite normalised to internal standard is shown.
(XLSX)
