# Supplementary material for: Time-course metabolic profiling in alfalfa leaves under Phoma medicaginis infection
Source: PLoS One. 2018 Oct 29;13(10):e0206641. doi: 10.1371/journal.pone.0206641 (PMC6205659; doi:10.1371/journal.pone.0206641)
Supplement: S1 Table — Relevant pathways based on MetaboAnalyst 4.0 analysis. (DOCX) [file pone.0206641.s001.docx]

S1 Table Relevant pathways of 16 significant regulated metabolites

| No. | Pathway Name | Total | | Hits | | | -log(*p*) | | Impact | | Details |
| --- | --- | --- | --- | --- | --- | --- | --- | --- | --- | --- | --- |
| 1 | [Glucosinolate biosynthesis](http://www.metaboanalyst.ca/faces/Secure/pathway/ResultView.xhtml) | 54 | | Methionine; Leucine; Isoleucine | | | 7.524 | | 0.0 | | Ath00966 |
| 2 | [Cysteine and methionine metabolism](http://www.metaboanalyst.ca/faces/Secure/pathway/ResultView.xhtml) | 34 | | Methionine | | | 7.38 | | 0.16 | | Ath00270 |
| 3 | [Aminoacyl-tRNA biosynthesis](http://www.metaboanalyst.ca/faces/Secure/pathway/ResultView.xhtml) | [67](http://www.metaboanalyst.ca/faces/Secure/pathway/ResultView.xhtml) | | Glycine; Methionine; Lysine; Leucine; Isoleucine; Tyrosine | | | 7.14 | | 0.0 | | [ath00970](http://www.genome.jp/kegg-bin/show_pathway?ath00970) |
| 4 | [Alanine, aspartate and glutamate metabolism](http://www.metaboanalyst.ca/faces/Secure/pathway/ResultView.xhtml) | [22](http://www.metaboanalyst.ca/faces/Secure/pathway/ResultView.xhtml) | | Gamma-Aminobutyric acid | | | 6.60 | | 0.0 | | ath00250 |
| 5 | [Arginine and proline metabolism](http://www.metaboanalyst.ca/faces/Secure/pathway/ResultView.xhtml) | [38](http://www.metaboanalyst.ca/faces/Secure/pathway/ResultView.xhtml) | | Gamma-Aminobutyric acid | | | 6.60 | | 0.0 | | [ath00330](http://www.genome.jp/kegg-bin/show_pathway?ath00330) |
| 6 | [Butanoate metabolism](http://www.metaboanalyst.ca/faces/Secure/pathway/ResultView.xhtml) | [18](http://www.metaboanalyst.ca/faces/Secure/pathway/ResultView.xhtml) | | Gamma-Aminobutyric acid | | | 6.60 | | 0.0 | | [ath00650](http://www.genome.jp/kegg-bin/show_pathway?ath00650) |
| 7 | [Fatty acid elongation in mitochondria](http://www.metaboanalyst.ca/faces/Secure/pathway/ResultView.xhtml) | [13](http://www.metaboanalyst.ca/faces/Secure/pathway/ResultView.xhtml) | | Palmitic acid | | | 5.98 | | 0.0 | | [ath00062](http://www.genome.jp/kegg-bin/show_pathway?ath00062) |
| 8 | [Fatty acid degradation](http://www.metaboanalyst.ca/MetaboAnalyst/faces/Secure/pathway/ResultView.xhtml) | [34](http://www.metaboanalyst.ca/faces/Secure/pathway/ResultView.xhtml) | | Palmitic acid | | | 5.98 | | 0.0 | | [ath00071](http://www.genome.jp/kegg-bin/show_pathway?ath00071) |
| 9 | [Fatty acid biosynthesis](http://www.metaboanalyst.ca/faces/Secure/pathway/ResultView.xhtml) | [49](http://www.metaboanalyst.ca/faces/Secure/pathway/ResultView.xhtml) | | Stearic acid; Palmitic acid | | | 5.74 | | 0.0 | | [ath00061](http://www.genome.jp/kegg-bin/show_pathway?ath00061) |
| 10 | [Biosynthesis of unsaturated fatty acids](http://www.metaboanalyst.ca/faces/Secure/pathway/ResultView.xhtml) | [42](http://www.metaboanalyst.ca/faces/Secure/pathway/ResultView.xhtml) | | Palmitic acid; Stearic acid | | | 5.74 | | 0.0 | | [ath01040](http://www.genome.jp/kegg-bin/show_pathway?ath01040) |
| 11 | [Glyoxylate and dicarboxylate metabolism](http://www.metaboanalyst.ca/faces/Secure/pathway/ResultView.xhtml) | [17](http://www.metaboanalyst.ca/faces/Secure/pathway/ResultView.xhtml) | | Malate | | | 5.36 | | 0.16 | | ath00630 |
| 12 | [Pyruvate metabolism](http://www.metaboanalyst.ca/faces/Secure/pathway/ResultView.xhtml) | [21](http://www.metaboanalyst.ca/faces/Secure/pathway/ResultView.xhtml) | | Malate | | | 5.36 | | 0.09 | | [ath00620](http://www.genome.jp/kegg-bin/show_pathway?ath00620) |
| 13 | [Citrate cycle (TCA cycle)](http://www.metaboanalyst.ca/faces/Secure/pathway/ResultView.xhtml) | [20](http://www.metaboanalyst.ca/faces/Secure/pathway/ResultView.xhtml) | | Malate | | | 5.3657 | | 0.03 | | [ath00020](http://www.genome.jp/kegg-bin/show_pathway?ath00020) |
| 14 | [Carbon fixation in photosynthetic organisms](http://www.metaboanalyst.ca/faces/Secure/pathway/ResultView.xhtml) | [21](http://www.metaboanalyst.ca/faces/Secure/pathway/ResultView.xhtml) | | Malate | | | 5.36 | | 0.0 | | [ath00710](http://www.genome.jp/kegg-bin/show_pathway?ath00710) |
| 15 | [Galactose metabolism](http://www.metaboanalyst.ca/faces/Secure/pathway/ResultView.xhtml) | [26](http://www.metaboanalyst.ca/faces/Secure/pathway/ResultView.xhtml) | | Sucrose; Myoinositol | | | 4.57 | | 0.04 | | [ath00052](http://www.genome.jp/kegg-bin/show_pathway?ath00052) |
| NO. | Pathway | | Total | | Hits | -log(*p*) | | Impact | | Details | |
| 16 | [Ascorbate and aldarate metabolism](http://www.metaboanalyst.ca/faces/Secure/pathway/ResultView.xhtml) | | [15](http://www.metaboanalyst.ca/faces/Secure/pathway/ResultView.xhtml) | | Myoinositol | 4.17 | | 0.0 | | [ath00053](http://www.genome.jp/kegg-bin/show_pathway?ath00053) | |
| 17 | [Inositol phosphate metabolism](http://www.metaboanalyst.ca/faces/Secure/pathway/ResultView.xhtml) | | [24](http://www.metaboanalyst.ca/faces/Secure/pathway/ResultView.xhtml) | | Myoinositol; Inositol 1,3,4,5,6-pentakisphosphate | 4.04 | | 0.25 | | [ath00562](http://www.genome.jp/kegg-bin/show_pathway?ath00562) | |
| 18 | [Glutathione metabolism](http://www.metaboanalyst.ca/faces/Secure/pathway/ResultView.xhtml) | | [26](http://www.metaboanalyst.ca/faces/Secure/pathway/ResultView.xhtml) | | Glycine; 5-oxoproline | 3.85 | | 0.01 | | [ath00480](http://www.genome.jp/kegg-bin/show_pathway?ath00480) | |
| 19 | [Valine, leucine and isoleucine biosynthesis](http://www.metaboanalyst.ca/faces/Secure/pathway/ResultView.xhtml) | | [26](http://www.metaboanalyst.ca/faces/Secure/pathway/ResultView.xhtml) | | Leucine; Isoleucine | 3.61 | | 0.02 | | ath00290 | |
| 20 | [Valine, leucine and isoleucine degradation](http://www.metaboanalyst.ca/faces/Secure/pathway/ResultView.xhtml) | | [34](http://www.metaboanalyst.ca/faces/Secure/pathway/ResultView.xhtml) | | Leucine; Isoleucine | 3.61 | | 0.0 | | [ath00280](http://www.genome.jp/kegg-bin/show_pathway?ath00280) | |
| 21 | [Isoquinoline alkaloid biosynthesis](http://www.metaboanalyst.ca/faces/Secure/pathway/ResultView.xhtml) | | 6 | | Leucine; Isoleucine | 3.41 | | 0.5 | | [ath00950](http://www.genome.jp/kegg-bin/show_pathway?ath00950) | |
| 22 | [Tyrosine metabolism](http://www.metaboanalyst.ca/faces/Secure/pathway/ResultView.xhtml) | | 18 | | Tyrosine | 3.41 | | 0.27 | | [ath0035](http://www.genome.jp/kegg-bin/show_pathway?ath00350) | |
| 23 | [Ubiquinone and other terpenoid-quinone biosynthesis](http://www.metaboanalyst.ca/faces/Secure/pathway/ResultView.xhtml) | | [23](http://www.metaboanalyst.ca/faces/Secure/pathway/ResultView.xhtml) | | Tyrosine | 3.41 | | 0.0 | | [ath00130](http://www.genome.jp/kegg-bin/show_pathway?ath00130) | |
| 24 | [Phenylalanine, tyrosine and tryptophan biosynthesis](http://www.metaboanalyst.ca/faces/Secure/pathway/ResultView.xhtml) | | [21](http://www.metaboanalyst.ca/faces/Secure/pathway/ResultView.xhtml) | | Tyrosine | 3.41 | | 0.0 | | [ath00400](http://www.genome.jp/kegg-bin/show_pathway?ath00400) | |
| 25 | [Glycine, serine and threonine metabolism](http://www.metaboanalyst.ca/faces/Secure/pathway/ResultView.xhtml) | | [30](http://www.metaboanalyst.ca/faces/Secure/pathway/ResultView.xhtml) | | Glycine | 3.10 | | 0.22 | | [ath00260](http://www.genome.jp/kegg-bin/show_pathway?ath00260) | |
| 26 | [Cyanoamino acid metabolism](http://www.metaboanalyst.ca/faces/Secure/pathway/ResultView.xhtml) | | [11](http://www.metaboanalyst.ca/faces/Secure/pathway/ResultView.xhtml) | | Glycine | 3.10 | | 0.0 | | [ath00460](http://www.genome.jp/kegg-bin/show_pathway?ath00460) | |
| 27 | [Nitrogen metabolism](http://www.metaboanalyst.ca/faces/Secure/pathway/ResultView.xhtml) | | [15](http://www.metaboanalyst.ca/faces/Secure/pathway/ResultView.xhtml) | | Glycine | 3.10 | | 0.0 | | [ath00910](http://www.genome.jp/kegg-bin/show_pathway?ath00910) | |
| 28 | [Starch and sucrose metabolism](http://www.metaboanalyst.ca/faces/Secure/pathway/ResultView.xhtml) | | [30](http://www.metaboanalyst.ca/faces/Secure/pathway/ResultView.xhtml) | | Sucrose | 2.64 | | 0.0226 | | [ath00500](http://www.genome.jp/kegg-bin/show_pathway?ath00500) | |
| 29 | [Lysine biosynthesis](http://www.metaboanalyst.ca/faces/Secure/pathway/ResultView.xhtml) | | [10](http://www.metaboanalyst.ca/faces/Secure/pathway/ResultView.xhtml) | | Lysine | 2.18 | | 0.07 | | [ath00300](http://www.genome.jp/kegg-bin/show_pathway?ath00300) | |
| 30 | [Lysine degradation](http://www.metaboanalyst.ca/faces/Secure/pathway/ResultView.xhtml) | | [17](http://www.metaboanalyst.ca/faces/Secure/pathway/ResultView.xhtml) | | Lysine | 2.18 | | 0.0 | | ath00310 | |

Total is the total number of compounds in the pathway; The hits = matched metabolites based on the uploaded data of 16 significant regulated metabolites from the control and inoculated groups at each time, respectively; The p is calculated with the enrichment analysis; Impact = pathway impact value calculated from pathway topology analysis. Details includes the number and links of the pathway in Kyoto Encyclopedia of Genes Genomes（Kegg）.
